# THE IMPACT OF NEIGHBORHOOD CONNECTEDNESS ON LIFE SATISFACTION AND SPIRITUAL EXPERIENCES ACROSS THREE AGE GROUPS.

**DOI:** 10.1093/geroni/igz038.3122

**Published:** 2019-11-08

**Authors:** Stephen J Fogle, Christopher M Kelly

**Affiliations:** University of Nebraska - Omaha, Omaha, Nebraska, United States

## Abstract

This study operationalized the third dimension of Rowe & Khan’s Successful Aging model, social engagement, as neighborhood connectedness. We examined 2820 older adults in the MIDUS III dataset to assess the impact of neighborhood connectedness on life satisfaction and daily spiritual experiences. A composite scale for neighborhood connectedness (Cronbach = .745) was created. Linear regression analysis was undertaken for life satisfaction on daily spiritual experience, neighborhood connectedness, neighborhood environment and age controlling for gender, co-habitation, income, and disability. Regression analysis was also conducted for daily spiritual experience on the same variables. Analysis for each outcome variable was run three times to explore changes across three age groups of older adults (55-69, 70-85, and 86-100). Results of regression analysis found frequency of daily spiritual experience was a substantial and significant predictor of life satisfaction for all age groups (β= .211, β= .191, β= .208) Additionally, regression analysis revealed a higher level of neighborhood connectedness was the most powerful predictor of daily spiritual experience across all age groups (β= .329, β= .312, β= .327) This study demonstrates the applicability of operationalizing the Successful Aging model’s social engagement dimension as neighborhood connectedness. This study also contributes evidence of the impact of daily spiritual experience on life satisfaction. Finally, the study supplies promising new evidence linking neighborhood connectedness with spiritual well-being.

